# Enhancing nutritional knowledge and self-regulation among adolescents: efficacy of a multifaceted food literacy intervention

**DOI:** 10.3389/fpsyg.2024.1405414

**Published:** 2024-09-13

**Authors:** Stefania Mancone, Stefano Corrado, Beatrice Tosti, Giuseppe Spica, Francesco Di Siena, Francesco Misiti, Pierluigi Diotaiuti

**Affiliations:** Department of Human, Social, and Health Sciences, University of Cassino, Cassino, Italy

**Keywords:** adolescent health literacy, nutritional education, eating habit intervention, self-regulation in diet, BMI improvement strategies, digital learning tools, emotional eating reduction, food knowledge enhancement

## Abstract

This health literacy intervention study, conducted on adolescent students, aimed to evaluate the impact of a comprehensive educational program on promoting healthy eating habits. The intervention sought to enhance adolescents’ knowledge about nutrition, foster self-regulation skills, and ultimately improve their overall health, including their body mass index (BMI). Through a multi-component approach that combined theoretical learning with practical activities and the integration of digital tools such as the MyFitnessPal app, the study targeted improvements in food literacy, which encompasses nutrition knowledge, food label interpretation skills, and cooking abilities. These elements are critical in influencing adolescents’ food choices and eating behaviors, with a focus on increasing the consumption of fruits and vegetables while reducing the intake of fast food and processed snacks. The intervention was structured around a series of educational workshops and interactive sessions, facilitated by professionals experienced in nutrition. These sessions covered topics such as food composition, the importance of a balanced diet, and strategies for avoiding excessive consumption of processed and high-energy-density foods. A distinctive feature of the program was its use of digital tools to enhance engagement and allow for the practical application of learned concepts through food tracking and activity monitoring. Preliminary results indicate positive outcomes in terms of increased food knowledge and improved dietary habits among participants. Notably, there was a significant improvement in adolescents’ BMI, highlighting the potential of food literacy interventions to contribute to better physical health outcomes. The study underscores the importance of adopting multidisciplinary and technology-enhanced approaches in educational programs aimed at promoting healthy eating among adolescents. The study’s findings suggest that well-structured food education programs, tailored to address the specific needs of adolescents, can facilitate positive changes in eating behavior. This highlights the critical role of food literacy in adolescents’ health and wellbeing and points to the need for continued investment in research and development to optimize intervention strategies. The integration of digital technologies and a focus on self-regulation strategies are identified as promising avenues for future research and educational practice, reinforcing the call for innovative investments in food education and health promotion among the youth.

## 1 Introduction

Health literacy among adolescents is critical for empowering them to make informed decisions about their health, encompassing not only knowledge of health topics but also the ability to effectively analyze and apply this information in various contexts (Koca and Arkan, 2020; Sørensen et al., 2012). This skill set directly impacts their health behaviors, such as dietary choices and physical activity, which are crucial during the developmental phase of adolescence (Schulenkorf et al., 2022; Cavicchiolo et al., 2022; Tsubakita et al., 2020; Guo et al., 2018).

Research has shown that health literacy among adolescents is linked to various health outcomes, such as medication adherence, mental health, and obesity (Ramos et al., 2023; Malloy-Weir et al., 2016; Murphy et al., 2010). Low levels of health literacy have been associated with negative health behaviors, poorer health status, and an increased risk of overweight and obesity (Guo et al., 2020). Health literacy plays a significant role in promoting positive health behaviors and empowering adolescents to take control of their health (Taba et al., 2022; Mallia et al., 2020; Awofeso et al., 2017).

Food literacy, which includes nutrition knowledge, food label interpretation skills, and cooking abilities, is a crucial aspect of adolescent health and wellbeing. Studies have highlighted that higher levels of food literacy significantly influence adolescents’ food choices and eating behaviors, leading to increased consumption of fruits and vegetables and reduced intake of fast food and processed snacks (Murad et al., 2021; Koca and Arkan, 2020; Vaitkeviciute et al., 2014).

Interventions aimed at enhancing food literacy among adolescents have shown promising results in improving dietary intake and overall health. School-based culinary programs and nutrition education initiatives have successfully increased adolescents’ food skills, nutrition knowledge, and cooking confidence, leading to healthier eating patterns (Labbé et al., 2023; Ruiz et al., 2021; LeRouge et al., 2019). Social media literacy programs have proven effective in reducing risk factors for eating disorders among adolescents, particularly by addressing body dissatisfaction and disordered eating behaviors (Muth et al., 2022; Agam-Bitton et al., 2018; McLean et al., 2017).

The link between health literacy and dietary behaviors in adolescents is well-documented, with higher levels of health literacy being associated with better preventive and healthy eating behaviors (Sarhan et al., 2022; Bektaş et al., 2021). Adolescents with higher health literacy are more aware of unhealthy foods and more likely to develop healthier eating habits. Also, parental health literacy has been correlated with adolescent health behaviors, highlighting the crucial role of parental involvement and support in promoting healthy eating habits among adolescents (Champion et al., 2023).

Adolescents’ eating habits are critical to their health and wellbeing as this life stage is marked by increased nutritional needs due to rapid growth and development (Yoshida-Montezuma et al., 2020). Poor dietary choices during adolescence can lead to serious health issues such as obesity, glucose intolerance, high blood pressure, and dyslipidemia (Kim and Lim, 2019).

Nutritional education and targeted interventions have proven effective in improving adolescents’ eating behaviors and nutrition knowledge (Bektaş et al., 2021; Qutteina et al., 2021; Raikar et al., 2020; Wang et al., 2015). Equipping adolescents with the information and skills necessary to make healthy food choices can significantly improve their long-term health outcomes (Grover and Choudhary, 2017; Yang et al., 2017; LeRouge et al., 2019; Depboylu, 2023).

However, many adolescents have limited food literacy skills, which can hinder their ability to plan, manage, select, prepare, and consume healthy foods (Bereznay et al., 2019). Interventions focused on promoting health literacy related to eating disorders in non-clinical settings have the potential to reduce the disease burden associated with common eating disorders in adolescence (Patton et al., 2008).

In this context, our study aims to evaluate the effectiveness of a targeted health literacy intervention designed to improve adolescents’ food literacy and promote healthier eating habits. The intervention focuses on enhancing their understanding of nutrition, improving their self-regulation skills, and ultimately contributing to better overall health outcomes, including a reduction in body mass index (BMI). The health literacy intervention implemented in this study combines educational workshops, interactive sessions, and the use of digital tools such as the MyFitnessPal app to enhance food literacy, which encompasses nutrition knowledge, food label interpretation skills, and cooking abilities.

## 2 Materials and methods

The study methodology involved the use of a health literacy intervention on healthy eating conducted on 200 adolescent students. The intervention employed was a multifaceted health literacy program specifically designed to enhance food literacy among adolescents and informed by Velardo (2015) who emphasizes the importance of health literacy, nutrition literacy, and food literacy when designing interventions aimed at improving dietary behaviors, with each playing a role in shaping individuals’ dietary behaviors and overall health outcomes. Food literacy, for example, requires a base of nutrition literacy but also includes practical skills such as cooking and an understanding of where food comes from. Velardo argues that effective interventions should be tailored to address the specific components of food literacy, recognizing that improving food-related knowledge alone is insufficient without also developing practical skills and critical thinking about food choices. Our program integrated educational workshops, practical exercises, and digital tools such as the MyFitnessPal app to improve participants’ knowledge of nutrition, food label interpretation, and self-regulation skills. The intervention was theoretically grounded in social cognitive theory (Bandura, 1986), which emphasizes the role of observational learning, social influence, and self-efficacy in behavior change. According to this theory, individuals learn new behaviors not only through direct experience but also by observing the actions of others and the resulting consequences. This approach is particularly relevant for adolescents, who are strongly influenced by their peers and authority figures. The intervention also incorporated elements of self-determination theory (Deci and Ryan, 1985; Ryan and Deci, 2017; Ryan and Deci, 2000), which focuses on the importance of fulfilling three fundamental psychological needs: autonomy, competence, and relatedness. By promoting these aspects, the intervention aimed to intrinsically motivate adolescents to make healthier food choices by feeling competent in their dietary decisions and supported by their environment.

This methodology was aimed at evaluating the effects of such an intervention on food knowledge, eating habits, and the overall health of adolescents. The method included the use of specific tools to assess these effects and collected quantitative and qualitative data. The study sample consisted of adolescent students selected from various high schools. The health literacy intervention was based on a series of educational workshops, interactive sessions, and educational materials aimed at improving students’ knowledge and eating habits. Before full implementation, the intervention underwent a pilot phase to assess its feasibility and preliminary effectiveness. During this phase, feedback was collected from participants to identify potential issues and opportunities for improvement. Adjustments were made to the educational content and delivery methods to ensure they were appropriately tailored to the target group. During the main intervention, a process evaluation was conducted to ensure fidelity and consistency in its delivery. This process included monitoring the sessions, reviewing practical activities, and tracking participants’ use of digital tools. We primarily focused on enhancing self-regulation skills related to dietary habits, such as goal-setting, planning, and resisting temptations. However, we acknowledge that self-regulation is distinct from practical skills such as cooking or food label interpretation. While our educational workshops did include components aimed at improving these practical skills, the study did not include direct assessments of these competencies. The study was approved by the Institutional Review Board, and all participants (and their legal guardians, in the case of minors) provided written informed consent before participation.

### 2.1 Participant selection

Participants were selected through voluntary sampling, with inclusion criteria that included being aged between 14 and 18 years and attending high schools. Exclusion criteria included the presence of diagnosed eating disorders and participation in previous food education programs in the last year. Participant selection was carried out by considering a sample of 200 adolescent students. The sample comprised boys and girls from various schools across the Lazio Region, including both urban and rural areas, to ensure a diverse representation of socio-economic and cultural backgrounds. Participants were selected from schools in urban centers as well as from more rural settings to capture a broad spectrum of experiences and contexts. Socio-economic status was assessed based on parental education and occupation, household income, and access to resources, with the aim of including students from low, middle, and high socio-economic backgrounds. The selection was made randomly among eligible students, thus ensuring the representativeness of the sample in the study context. To further enhance the diversity and inclusivity of our participant recruitment process, we employed multiple strategies to reach a broader demographic. This included leveraging social media platforms and school newsletters to disseminate information about our study, as well as engaging directly with community organizations that work closely with adolescents. Efforts were made to ensure our recruitment message was culturally sensitive and accessible, using clear, jargon-free language to appeal to both adolescents and their guardians. We hosted informational sessions at participating schools, providing a forum for potential participants and their parents to ask questions and express concerns. These sessions not only served to inform but also to build trust within the community, an essential element in encouraging participation. Our approach aimed at not only fulfilling our recruitment targets but also ensuring a sample that accurately reflects the diversity of dietary habits and health literacy levels among adolescents in the region. Following Table 1 provides a detailed breakdown of the sample’s demographic and socio-economic characteristics. This table includes information on the distribution of participants by gender, geographical location (urban vs. rural), and socio-economic status, as measured by parental education, occupation, and household income. The table provides details on the number of schools and classes involved in the study, highlighting the breadth of the sample across different educational settings.

**Table 1:** Demographics and school data.

Variable	Category	Number of Participants (*n*)	Percentage (%)
Gender	Male	89	44.5%
	Female	111	55.5%
Geographical location	Urban	95	47.5%
	Rural	105	52.5%
Socio-economic status	Low	45	22.5%
	Middle	65	32.5%
	High	90	45.0%
Parental education	Primary education	40	20.0%
	Secondary education	85	42.5%
	Tertiary education	75	37.5%
Parental occupation	Unskilled labor	36	18.0%
	Skilled labor	65	32.5%
	Professional	99	49.5%
Number of schools		12	
Number of classes		45	

### 2.2 The health literacy intervention

The health literacy intervention procedure typically involves several key steps designed to enhance individuals’ understanding and skills regarding health information, particularly in the context of nutrition and healthy eating. Here is a summarized version of a typical procedure:

1. Pre-assessment: The procedure often starts with a pre-assessment phase, where participants’ baseline knowledge and attitudes toward nutrition and healthy eating are evaluated. This could be done through structured questionnaires or interviews to establish a starting point for measuring the intervention’s effectiveness.
2. Design of educational content: Based on the pre-assessment results, educational materials and sessions are tailored to address the specific needs and gaps in knowledge identified. This content usually covers topics such as the basics of a balanced diet, the role of different nutrients in the body, how to read food labels, and strategies for making healthier food choices.
3. Delivery of educational sessions: The core of the intervention involves delivering the designed educational content. This is often done through interactive sessions that could include presentations, workshops, group discussions, and practical activities. These sessions are usually led by health professionals or trained educators who specialize in nutrition and dietetics.
4. Practical activities: To reinforce learning, participants might engage in practical activities such as meal planning exercises, cooking demonstrations, and grocery shopping tours. These activities help participants apply what they have learned in real-life settings.
5. Post-intervention assessment: After the educational sessions, a post-intervention assessment is conducted to evaluate the impact of the health literacy intervention. This involves measuring changes in participants’ knowledge, attitudes, and behaviors related to nutrition and healthy eating. The same tools used in the pre-assessment phase can be employed to facilitate a comparative analysis.
6. Follow-up: Depending on the intervention’s design, a follow-up phase might be included to assess the long-term effectiveness of the program. This could involve additional surveys or interviews conducted several months after the intervention.
7. Analysis and reporting: The final step involves analyzing the data collected during the pre- and post-assessment phases to determine the intervention’s effectiveness. The findings are then reported to stakeholders, which can include participants, school officials, healthcare providers, and the wider community.

This procedure is designed to be flexible and adaptable to different contexts and target populations. The ultimate goal is to empower participants with the knowledge and skills needed to make informed decisions about their dietary habits and overall health.

### 2.3 The intervention

The health literacy intervention procedure involved several phases. Initially, an initial assessment of the adolescents’ food knowledge and eating self-regulatory skills was conducted through a structured questionnaire. Then, an educational intervention focusing on promoting healthy eating was carried out. This intervention was conducted through health education sessions led by professionals experienced in the field of nutrition. The sessions included information on food composition, planning a balanced diet, and the importance of nutrition for health. Finally, a post-intervention evaluation was conducted to assess the effectiveness of the health literacy intervention on the adolescents’ food knowledge. The intervention consisted of a 6-week health literacy program, with weekly meetings of approximately 1 h each. Each session was structured to cover different aspects of nutrition and healthy eating, including the principles of a balanced diet, the importance of various nutrients, strategies to avoid excessive consumption of processed and high-energy-density foods, and how to read and interpret food labels. Following the post-intervention assessment (3 months after the conclusion of the seminars), a final group feedback session was run to discuss the adolescents’ progress, challenges encountered, and strategies for sustaining their healthy eating habits.

The intervention was run by a team of four experts and involved, respectively, a dietitian creating personalized nutrition plans, a nutritionist offering educational workshops on healthy eating, a health educator developing engaging activities to promote lifestyle changes, and a psychologist supporting students in overcoming barriers to healthy eating. This multidisciplinary approach ensured a comprehensive educational experience, addressing both the nutritional knowledge and emotional aspects of adopting healthier eating habits.

### 2.4 Educational materials

Educational materials included informational brochures, educational videos, and mobile applications for diet tracking. Students also received food diaries to record their daily diet. A diverse array of formats was utilized to cater to different learning preferences among adolescents. This included interactive, multimedia content on nutrition and health for a hands-on approach to learning about food groups and nutritional values and podcasts featuring discussions with nutrition experts and peers sharing their healthy eating journeys. These materials were designed not only to educate but also to inspire and motivate adolescents by showcasing relatable success stories and practical tips for adopting healthier eating habits. Among the applications recommended for adolescents, the following were presented and utilized: (1) App MyFitnessPal: This app is widely used for tracking nutrition and physical activity. It offers an extensive database of foods, including many products available on the Italian market, allowing users to monitor their caloric intake and nutrient consumption. (2) App Yazio: Another app for diet and exercise tracking, Yazio provides personalized meal plans and challenges to encourage healthy lifestyles. This app is also accessible in Italy and can be an excellent educational tool for adolescents. (3) YouTube Italia Nutrizione: This YouTube channel offers educational videos on various aspects of nutrition in an easily accessible and engaging format for adolescents. (4) Fooducate: Although not specifically Italian, Fooducate is an app that allows users to scan the barcodes of food products to receive detailed information about their nutritional quality, suggestions for healthier alternatives, and much more. It can be used to teach adolescents to make informed food choices.

### 2.5 Evaluation methods

The evaluation tools used in the study were selected based on the goal of assessing the effect of the health literacy intervention on healthy eating and self-regulatory skills among adolescents. To assess the impact of health literacy interventions, we used the Italian version of the Nutritional Literacy Scale, which measures knowledge and skills related to nutrition (Vettori et al., 2021; Gibbs and Chapman-Novakofski, 2012). The tool was developed to assess nutrition literacy in terms of nutrition knowledge and skills in making food choices. The instrument has six subscales, with 64 items: (1) ‘Nutrition & Health’ which measures reading comprehension of the summarized Dietary Guidelines for Italians; (2) ‘Energy Sources in Food’ which measures knowledge of the macronutrient sources in food; (3) ‘Household Food Measurement’ which measures identification of recommended portions; (4) ‘Food Label and Numeracy’ which measures ability to apply information obtained from the nutrition facts panel; (5) ‘Food Groups’ which measures ability to classify foods by nutrition category, and (6) ‘Consumer Skills’ which measures ability to navigate food products to make healthy food choices. This tool allowed for the collection of quantitative data on the understanding and skills of adolescents in the field of nutrition. In the present study, the coefficient omega ranged from 0.78 to 0.80 for the six subscales.

In addition, an instrument focusing on the emotional compensation aspects of feeding was included in the evaluation protocol, that is, the Emotional Eating Scale (EES, Arnow et al., 1995); It. Valid. Lombardo and San Martini (2005) that includes 25 items assessing the desire to eat after negative emotions, and it is composed of three subscales: emotional eating after depression (EES-D), emotional eating after anxiety/confusion (EES-A), and emotional eating after anger (EES-R). Participants are asked to indicate the extent to which certain feelings lead them to feel an urge to eat (e.g., when they felt depressed, bored, angry, and agitated) on a five-point scale ranging from 1 (“no desire to eat”) to 5 (“an overwhelming urge to eat”). In the present study, the coefficient omega for the EES-D subscale was 0.79, for the EES-A, it was 0.75, and finally for the EES-R, it was 0.78.

Finally, a behavioral questionnaire tailored to Italian dietary patterns, the Tempest Self-Regulation Questionnaire for Eating (TESQ-E) (De Vet et al., 2014; It.valid.: Diotaiuti et al., 2022), has been used to evaluate changes in eating habits and eating self-regulation. The instrument assesses self-regulation and self-control strategies to counteract the desire and temptation to eat unhealthy food and to choose healthy foods. It includes 24 items, which are to be answered using a five-point scale, ranging from 1 (“never”) to 5 (“always”). The instrument is organized into six self-regulation strategies [first-order factors: avoidance of temptations (four items); controlling temptations (four items); distraction (four items); suppression (four items); goal and rule setting (four items); and goal deliberation (four items)] which loaded on three general self-regulation approaches (second-order factors: strategies that directly address temptations; strategies that address the meaning of temptations; and strategies that directly address the objectives) (Stok et al., 2013). In the present study, the coefficient omega for the Addressing directly Temptations (ADT) subscale was 0.82, for the Addressing Psychological Meaning of Temptations (APM), it was 0.81, and finally for the Addressing Goals (AG) subscale, it was 0.83.

Students were encouraged to use the digital food tracking app, MyFitnessPal, to track daily food intake, including details on macro- and micronutrients, allowing users to easily record meals consumed and physical activities performed, helping them better understand their daily caloric balance (calories consumed vs. calories burned). Health parameters were also collected before the intervention, at its conclusion, and after 3 months. These included body weight and height to calculate participants’ body mass index (BMI).

### 2.6 Statistical analysis

To analyze the results of the intervention study on food literacy among adolescents, the following statistical analyses were conducted using the statistical software SPSS v. 26:

1. Descriptive analysis: to provide a summary of the sample characteristics, including age, gender, initial BMI, and levels of food knowledge. This includes calculations of means, standard deviations, frequencies, and percentages.
2. Paired Student’s *t*-test: used to compare pre- and post-intervention scores on continuous variables (such as food knowledge and BMI) to assess whether there have been significant improvements due to the intervention.
3. Repeated measures analysis of variance (ANOVA): to examine differences in pre-intervention, post-intervention, and follow-up scores among study groups, if applicable. This can help to identify whether changes remain significant over time. Friedman’s non-parametric test was used if data dо not follow a normal distribution.
4. Correlation analysis: to explore relationships between variables, such as the relationship between food knowledge levels and changes in BMI, or between self-regulation strategies and the reduction of emotional eating.
5. Multiple logistic or linear regressions: to assess the impact of multiple variables (such as age, gender, and initial level of food knowledge) on the outcomes of the intervention (e.g., BMI improvement or changes in eating habits).
6. Wilcoxon rank sum analysis: a non-parametric test used if the data dо not follow a normal distribution, to compare median scores pre- and post-intervention on variables such as food knowledge or emotional eating.

## 3 Results

As reported in Table 2, the mean age of participants was approximately 17 years, with a small standard deviation, indicating a relatively homogenous group in terms of age. The initial BMI had a mean of 23.64 with a wider standard deviation, suggesting variability in participants’ body mass index. The Nutritional Literacy (NLit) and Emotional Eating Scale (EES) scores also varied, indicating diverse levels of nutritional knowledge and emotional eating behaviors among participants. The TESQ scores, which measure self-regulation in eating, had a mean of 2.35, with a standard deviation indicating some variability in participants’ ability to regulate their eating behavior.

**Table 2:** Descriptive sample analysis.

Variable	Mean	STD	Min	25th Percentile	Median	75th Percentile	Max
Age	16.97	0.81	16	–-	17	–	18
BMI initial	23.64	4.44	16.44	21.04	23.01	25.14	44.29
NLit initial	49.08	2.70	39	48	50	51	52
EES initial	61.23	21.36	25	45.75	53.5	80	121
TESQ initial	2.35	0.74	1.00	1.88	2.43	2.85	4.03

According to Table 3, there were 89 male participants, constituting 44.5% of the sample, and 111 female participants, making up 55.5% of the sample. This distribution shows a slightly higher representation of female participants in the study.

**Table 3:** Gender distribution.

Gender	*n*.	Percentage (%)
Male	89	44.5
Female	111	55.5

Table 4 presents Spearman’s bivariate correlations for several variables, including body mass index (BMI), Nutritional Literacy (NLit), Emotional Eating after Depression (DEP), Emotional Eating after Anger (ANG), Emotional Eating after Anxiety (ANX), Emotional Eating Scale (EES), and several self-regulation strategies as measured by the Tempest Self-Regulation Questionnaire for Eating (TESQ) among others. The table provides correlation coefficients that measure the strength and direction of association between pairs of these variables, with **p < 0.01 indicating statistically significant correlations. Key findings from Table 3 include the following:

**Table 4:** Spearman’s bivariate correlations.

	BMI	NLit	DEP	ANG	ANX	EES	TEMP	MEAN	GOAL	TESQ
BMI	1									
NLit	0.021	1								
DEP	0.367**	−0.042	1							
ANG	0.272**	−0.032	0.843**	1						
ANX	0.253**	−0.016	0.806**	0.870**	1					
EES	0.298**	−0.014	0.927**	0.953**	0.940**	1				
TEMP	−0.329**	0.210^**^	−0.402**	−0.321**	−0.325**	−0.347**	1			
MEAN	−0.315**	0.218**	−0.309**	−0.275**	−0.220**	−0.263**	0.663**	1		
GOAL	−0.301**	0.247**	−0.418**	−0.346**	−0.336**	−0.363**	0.673**	0.778**	1	
TESQ	−0.339**	0.286**	−0.420**	−0.342**	−0.324**	−0.357**	0.860**	0.876**	0.917**	1

BMI, body mass index; NLit, nutritional literacy scale; DEP, emotional eating after depression; ANG, emotional eating after anger; ANX, emotional eating after anxiety; EES, emotional eating scale; TEMP, addressing directly temptations; MEAN, addressing psychological meaning of temptations; GOAL, addressing goals; TESQ, tempest self-regulation questionnaire for eating. **p < 0.01.

Significant positive correlations among emotional eating measures (DEP, ANG, ANX, and EES) indicate that higher levels of emotional eating in one category (e.g., depression) are associated with higher levels of emotional eating in other categories (e.g., anger and anxiety).

Negative correlations between Emotional Eating Measures and Self-Regulation Strategies (TEMP, MEAN, and GOAL) suggest that individuals who report higher use of self-regulation strategies to manage their eating behavior tend to report lower levels of emotional eating. This highlights the potential protective role of self-regulation skills against emotional eating behaviors.

BMI’s correlation with Emotional Eating and Self-Regulation Strategies is notable. BMI shows a positive correlation with emotional eating measures, indicating that higher BMI values are associated with higher levels of emotional eating. Conversely, BMI is negatively correlated with self-regulation strategies, suggesting that individuals with higher BMIs may utilize fewer self-regulation strategies in their eating behaviors, or that effective use of such strategies could be associated with lower BMI.

Nutritional Literacy (NLit) shows very low or insignificant correlations with most other variables, indicating that nutritional knowledge alone may not be strongly associated with emotional eating behaviors or the use of specific self-regulation strategies. This suggests that while knowledge is important, it might not directly influence these behaviors without the support of practical skills or interventions designed to change behaviors.

As reported in Table 5, the mean BMI at baseline was 23.64, with a slight decrease post-intervention (*M* = 23.45) and at follow-up (*M* = 23.04). There was a noticeable increase in mean nutrition literacy from baseline (*M* = 49.08) to post-intervention (*M* = 56.06) and slightly decreasing at follow-up (*M* = 54.42), indicating an improvement after the intervention. The mean EES score decreased from baseline (*M* = 61.23) to post-intervention (*M* = 59.26) and further at follow-up (*M* = 57.92), suggesting a reduction in emotional eating. The self-regulation questionnaire scores showed an increasing trend from baseline (*M* = 2.35) to post-intervention (*M* = 2.62) and follow-up (*M* = 2.75), indicating improved self-regulation skills over time.

**Table 5:** Changes in mean values of variables in the three conditions.

	Initial assessment		Post-intervention		Follow-up	
Variable	Mean	STD	Mean	STD	Mean	STD
BMI	23.64	4.44	23.45	4.25	23.04**	4.36
NLit	49.08	2.70	56.06**	3.81	54.42**	3.61
EES	61.23	21.36	59.26	17.77	57.92	16.86
TESQ	2.35	0.74	2.62**	0.55	2.75**	0.57

**p < 0.001.

The Shapiro–Wilk test results for normality indicated that the distributions of BMI, NLit, EES, and TESQ at baseline were not normally distributed (*p*-values <0.05), which suggests that non-parametric tests should be used for further analyses.

Given that the data dо not follow a normal distribution for most variables, we used the Wilcoxon test (a non-parametric alternative to the *t*-test) to compare pre- and post-intervention scores, which indicated significant changes for some variables.

The Wilcoxon signed-rank tests comparing baseline versus post-intervention and baseline versus follow-up measurements for body mass index (BMI), Nutrition Literacy (NLit), Emotional Eating Scale (EES), and Tempest Self-Regulation Questionnaire for Eating (TESQ) yielded the following *p*-values:

- BMI Changes. Baseline vs. Post-intervention: *p*-value <0.001; Baseline vs. Follow-up: *p*-value <0.001. The significant change in BMI indicates that the intervention had a marked effect on reducing BMI from pre-intervention values to post-intervention values. This suggests success in achieving the goal of improving body weight or body composition of the individuals involved.
- Nutrition Literacy (NLit) Changes. Baseline vs. Post-intervention: *p*-value <0.001; Baseline vs. Follow-up: *p*-value <0.001. An extremely low p-value indicates that there was significant growth in food knowledge following the intervention. This implies that the intervention was very effective in improving the understanding of nutritional topics among the participants.
- Emotional Eating Scale (EES) Changes. Baseline vs. Post-intervention: p-value = 0.71; Baseline vs. Follow-up: *p*-value = 0.34. The p-value does not indicate significant changes in emotional eating pre- and post-intervention. This suggests that, despite the intervention, there were no significant variations in emotional eating behaviors among the participants.
- Changes in the Tempest Self-Regulation Questionnaire for Eating (TESQ). Baseline vs. Post-intervention: *p*-value <0.001; Baseline vs. Follow-up: *p*-value <0.001. The p-value indicated significant improvements in individuals’ self-regulation skills regarding their eating behavior following the intervention.

These results suggested that the intervention significantly impacted BMI, Nutrition Literacy, and self-regulation skills (as measured by TESQ), with improvements evident both immediately post-intervention and at follow-up. However, changes in Emotional Eating (as measured by EES) were not statistically significant, indicating that the intervention may not have had a clear impact on emotional eating behaviors.

The Friedman test results clearly show that there were significant differences between the scores of all the considered variables (BMI, NLit, EES, and TESQ) at the three measurement times (pre-intervention, post-intervention, and follow-up) (see Table 6). This demonstrates the lasting impact of the intervention beyond the period immediately following its conclusion. For BMI and NLit, not only the immediate impact was confirmed but also the persistence over time of this effect on body mass index and nutritional knowledge. For EES, although there were no significant changes in the pre- and post-analysis, the Friedman test indicated that, considering the follow-up, there were significant changes over time (*p*-value <0.001). For TESQ, similarly, it was confirmed that the intervention had a substantial and lasting effect on improving food self-regulation skills.

**Table 6:** Friedman test results (pre, post, and follow-up).

Variable	Chi square	*P*-value
BMI	110.67	< 0.001
NLit	360.33	< 0.001
EES	36.44	< 0.001
TESQ	82.53	< 0.001

Table 7 provides correlation coefficients along with *p*-values, highlighting the strength and significance of the relationships between the variables.

**Table 7:** Spearman’s correlation analysis results.

Relation	Correlation coefficient	*P*-value
Baseline NLit vs. Changes in BMI	0.165	0.019
Baseline TESQ vs. Changes in EES	0.310	0.001

Baseline NLit vs. Changes in BMI: The correlation coefficient of 0.165 with a p-value of 0.019 suggests a statistically significant, albeit weak, positive relationship between initial nutritional literacy levels and changes in BMI. This indicates that higher baseline levels of nutritional literacy might be associated with greater changes in BMI, suggesting that a better understanding of nutrition could positively influence BMI outcomes to some extent.

Baseline TESQ vs. Changes in EES: A stronger correlation coefficient of 0.310 with a *p*-value of 0.001 demonstrates a statistically significant, moderate relationship between initial self-regulation skills (as measured by TESQ) and changes in emotional eating (as measured by EES). This suggests that individuals who initially report better self-regulation in eating are more likely to experience significant changes in their emotional eating behaviors. This could imply that enhancing self-regulation skills may be an effective strategy in addressing emotional eating.

The regression analysis in Table 8 highlights the nuanced dynamics between demographic factors, nutritional literacy, and their collective impact on BMI changes. The significance of nutritional literacy underscores the value of targeted nutritional education in influencing positive health outcomes. The lack of a significant gender effect suggests that, in this sample, gender does not have a meaningful impact on the intervention’s effectiveness regarding BMI changes. The coefficient for age is −0.228 with a statistically significant p-value of 0.002. This suggests that age negatively affects BMI changes, implying that older adolescents might experience smaller changes in BMI compared to younger ones. This could be due to various factors, including differences in metabolic rates, lifestyle choices, or responsiveness to the intervention based on age. The initial level of nutritional literacy has a coefficient of 0.120 with a p-value of less than 0.001, indicating a positive and statistically significant impact on BMI changes. This implies that higher initial nutritional literacy is associated with greater changes in BMI, reinforcing the importance of nutritional education as part of interventions aimed at BMI management.

**Table 8:** Results of multiple linear regression (changes in BMI).

Variable	Coefficient	Standard error	*t*-Statistic	*P*-value
(Intercept)	−2.148	0.828	−2.594	0.010
Age	−0.228	0.072	−3.145	0.002
Genre	−0.024	0.079	−0.301	0.764
NLit	0.120	0.022	5.457	< 0.001

## 4 Discussion

The food literacy intervention carried out has demonstrated the importance of combining different strategies to promote healthy nutrition among adolescents. Going deeper into the results, it is possible to better understand how these strategies have contributed to improving food knowledge, eating habits, self-regulation, and the overall health of adolescents.

The increase in food knowledge among adolescents is a key outcome of the intervention. By educating students on the principles of a balanced diet, the importance of various nutrients, and how to read and interpret food labels, the intervention has provided them with the necessary tools to make informed food choices. This aspect is fundamental as the ability to understand and apply nutritional information is an essential first step toward adopting healthy eating habits.

The evident result of the increase in food knowledge among the participating adolescents is not only an indicator of the success of the educational intervention but also a critical foundation for the adoption of informed food choices. In-depth knowledge about nutrients, the caloric values of foods, and the composition of healthy meals is essential to combat improper eating habits that can lead to long-term health problems, such as obesity and diseases related to diet (Lee et al., 2022; Liu et al., 2022).

The intervention highlighted how self-regulation practices can positively influence adolescents’ eating habits. Strategies such as avoiding temptations, setting dietary goals and rules, and reflecting on personal goals can help resist the temptations of unhealthy foods and prefer more nutritious options. This approach proved effective in promoting an increase in fruit and vegetable consumption and a reduction in the intake of fast food and processed snacks. The improvement in self-regulation in eating habits testifies to the effectiveness of the intervention in promoting effective coping strategies in the face of temptations of unhealthy foods and in portion management. Self-regulation skill is crucial for maintaining a balanced diet and managing body weight, especially in an era where adolescents are constantly exposed to unhealthy food stimuli through media and social environment (Diotaiuti et al. (2021a); Guzek et al., 2022; Guo et al., 2023).

The adoption of apps for tracking food and physical activity, such as MyFitnessPal, provided adolescents with a practical and interactive method to monitor their diet and caloric balance. This not only increased their awareness of their eating habits but also facilitated the management and optimization of nutrient and calorie intake. The use of digital technologies in food education offers significant potential to improve engagement and learning among adolescents (König et al., 2021).

The positive effect of the intervention on adolescents’ BMI underscores the importance of food literacy for overall health. An improved BMI reflects not just a better body composition but also a reduced risk of developing health problems related to diet, such as obesity and metabolic diseases. These results confirm that well-structured educational interventions can have a significant impact on the long-term health of adolescents (Reid et al., 2021; Taleb and Itani, 2021).

The improvement in body mass index (BMI) signals a direct positive impact of the intervention on the physical health of adolescents. BMI is a key indicator of health status and the risk of developing chronic diseases associated with overweight and obesity. An improvement in this parameter reflects not only a reduction in the intake of high-energy-density foods but also an increase in physical activity, highlighting the importance of a holistic approach to health that integrates nutrition and exercise (Koca and Arkan, 2020; Ruiz et al., 2021).

The interpretation of the results obtained from the food literacy intervention conducted on adolescents reveals a series of significant considerations that warrant detailed exploration to fully understand their impact and implications. These results lay the groundwork for further investigations and implementations in the field of food education and health promotion among adolescents. It is crucial to recognize that food literacy is not limited to the mere acquisition of knowledge but also includes the development of practical competence, such as reading food labels, preparing healthy meals, and effectively managing food resources (Ares et al., 2023; Li et al., 2022).

The study also indicated that there was no statistically significant change in Emotional Eating Scale (EES) scores following the eating literacy intervention among adolescents, suggesting that the intervention did not significantly alter emotional eating behaviors, which are often triggered by negative emotions such as anxiety, anger, or depression. Emotional eating, the act of consuming food in response to emotional rather than physical hunger, can lead to unhealthy eating patterns and potentially contribute to weight gain or the development of eating disorders (Erdem et al., 2023; Joseph et al., 2023; Xiang et al., 2023; Limbers and Summers, 2021).

The lack of significant change in emotional eating behaviors post-intervention highlights the complexity of addressing such behaviors through eating literacy programs alone. It underscores the necessity of integrating more targeted psychological or behavioral strategies within food literacy interventions to effectively manage emotional triggers for eating. This outcome emphasizes the importance of a multifaceted approach to food literacy interventions that not only educates adolescents about nutritional knowledge and self-regulation skills but also addresses the emotional aspects of eating (Bektas and Gürkan, 2023; Francis et al., 2023; Aloudah, 2021; Diotaiuti et al., 2020).

By incorporating strategies to better manage emotional responses that lead to eating, such interventions can contribute to more balanced eating behaviors and improve overall mental and physical health among adolescents. Future research should explore the incorporation of emotional regulation techniques and psychological support within eating literacy programs to enhance their effectiveness in reducing emotional eating among adolescents (Bui et al., 2021; Diotaiuti et al. (2021b); Woods-Townsend et al., 2021; Shriver et al., 2020).

The comparison of the results of the food literacy intervention among adolescents with those of previous studies highlights some interesting aspects and confirms the growing evidence on the effectiveness of educational interventions in promoting healthy eating habits in this critical age group.

Previous studies have already highlighted the importance of food education among adolescents, showing how targeted interventions can improve nutritional knowledge and positively influence food choices. For example, the effectiveness of school nutritional education programs in improving understanding and adoption of healthier diets has been documented in various research (Heikkilä et al., 2021; Noronha et al., 2020; Asakura et al., 2017; Egg et al., 2020). These programs, similar to the described intervention, have utilized multi-component educational approaches to influence both the knowledge and behavior of students.

A distinctive aspect of the intervention under examination is the intensive use of digital tools, such as the MyFitnessPal app, to promote self-regulation and the tracking of food intake and physical activity. This approach aligns with the research by Kloss and Bittlingmayer (2017), which emphasized the importance of eHealth literacy in promoting healthy eating behaviors among adolescents. The inclusion of digital technologies represents a relatively new feature in the field of food education and reflects the adaptation of interventions to the habits and preferences of modern adolescents.

The impact of the intervention on the BMI of adolescents echoes the findings of studies such as those reported by Jacob et al. (2021), which demonstrated how school-based interventions can have positive effects not only on knowledge and behavior but also on physical health measures such as BMI. This reinforces the idea that well-designed and well-executed interventions can have tangible beneficial effects on the physical health of adolescents.

However, it is important to note that, while the described intervention and similar studies show promising results, the literature also indicates the need for long-term interventions and follow-ups to maintain behavioral changes (Bailey et al., 2019; Diotaiuti et al. (2021c); Forray et al., 2021; Larose et al., 2023). Many studies underline the importance of continuous support, education, and environment to sustain healthy eating habits over time (Chaudhary et al., 2020; Medeiros et al., 2022; Nagy-Pénzes et al., 2022).

Our study highlights the importance of self-regulation in improving dietary behaviors among adolescents. However, we recognize the need to directly measure practical skills in future research. Practical skills, such as cooking and food preparation, are critical components of food literacy that were addressed in our intervention but not formally assessed. Future studies should incorporate these measures to provide a more comprehensive evaluation of food literacy interventions.

### 4.1 Limitations of the study

The limitations of a study are crucial for understanding the context in which the results can be interpreted and for guiding the direction of future research. Examining the food literacy intervention among adolescents, we can identify several limitations that deserve attention:

1. Study sample and generalizability: The sample might have been limited in size, geographical, or socio-economic diversity, which could affect the generalizability of the results. If the study was conducted in a specific area or with a homogeneous group of students, the results might not be applicable to other populations with different cultural, economic, or educational contexts.
2. Lack of a control group: The absence of a control group limits the ability to definitively attribute observed changes to the intervention alone. Future studies should incorporate a control group to strengthen the causal inferences regarding the effectiveness of health literacy interventions.
3. Duration of the intervention and follow-up: The duration of the intervention and the follow-up period might not have been long enough to assess the long-term effects on adopting healthy eating habits and maintaining changes in behavior and food knowledge. Long-term interventions and extended follow-ups are crucial for understanding the sustainability of behavioral changes.
4. Dependence on self-reporting: If the study relied on self-reported data from adolescents to track food intake, physical activity, or other behaviors, this could introduce a social desirability bias or inaccuracies due to faulty memory. These factors can lead to an overestimation or underestimation of behavioral changes.
5. Control of confounding variables: There might have been limitations in the ability to control all confounding variables that affect the eating habits and health of adolescents, such as peer influence, family support, access to healthy foods, and the school environment. The presence of these uncontrolled variables can obscure the interpretation of the results.
6. Measurement of outcomes: The metrics used to assess the outcomes of the intervention might not cover all relevant aspects of food literacy or eating behavior. For example, focusing on specific indicators such as BMI or fruit and vegetable intake might neglect other important dietary or lifestyle aspects.
7. Technology-based intervention: The use of digital tools, although innovative and engaging, might limit the participation of individuals with limited access to technology or less developed digital skills. This could create a disparity in the ability to benefit from the intervention.

Recognizing these limitations is fundamental not only for cautiously interpreting the study’s results but also for guiding the development of future food literacy interventions. Identifying these areas for improvement can contribute to designing more robust, inclusive, and representative studies that can provide more solid and generalizable evidence on the most effective strategies for promoting healthy eating habits among adolescents.

## 5 Implications for clinical practice and future research

The food literacy intervention among adolescents, despite its limitations, offers significant implications for both clinical practice and future research. These insights can guide the evolution of health education programs and inform the direction of subsequent investigations.

### 5.1 For clinical practice

1. Customization of interventions: the importance of tailoring food literacy interventions to the specific needs of different groups of adolescents, considering factors such as age, socio-economic context, culture, and technological predisposition. This can enhance the effectiveness of interventions and the adoption of healthy eating habits.
2. Integration of technology: clinical practice can benefit from integrating digital tools and apps for tracking nutrition and physical activity, encouraging adolescents to become more aware of their habits and to better manage their health.
3. Education of parents and caregivers: emphasizing the role of parents and caregivers in influencing adolescents’ eating habits, and promoting programs that also involve families in food education and health promotion.
4. Interdisciplinary collaboration: encouraging collaboration among health professionals, educators, and communities to develop and implement holistic, sustainable, and accessible food literacy programs for all adolescents.

### 5.2 For future research

1. Study of long-term interventions: conducting research on longer-duration interventions with extended follow-ups to assess the long-term impact on food literacy, eating habits, and health indicators.
2. Exploration of diversity and inclusivity: expanding research to include a greater diversity of participants, considering geographical, economic, and cultural variations, to improve the generalizability of the results.
3. Evaluation of new outcome metrics: developing and using assessment tools that capture a wide range of outcomes related to food literacy, including behavioral, psychological, and social changes, as well as physical health indicators.
4. Analysis of the impact of confounding variables: deepening the understanding of how factors such as peer influence, family and school environment, and access to healthy foods affect the effectiveness of food literacy interventions.
5. Use and impact of technology: further investigating the effect of digital tools on food education, exploring how different platforms and formats can be used to maximize engagement and learning among adolescents.
6. Future research should include a control group to provide a more robust evaluation of the intervention’s impact and enhance the generalizability of the findings.

Adopting a holistic, personalized, and collaborative approach can significantly improve the effectiveness of food literacy interventions, with the potential to positively impact public health in the long term.

## 6 Conclusion

The insights derived from this study emphasize the need for multidisciplinary approaches and the use of digital technologies in food education for adolescents. A well-designed program that combines theory with practical activities and the use of digital tools can significantly improve food knowledge, eating habits, and the overall health of adolescents. The importance of these findings lies in their applicability to future health education programs, indicating ways to further enhance the effectiveness of food literacy interventions.

The intervention has demonstrated that food education and literacy can have a significant impact on adolescents’ eating habits, improving their overall health and long-term wellbeing. These results highlight the importance of well-structured food education programs that are capable of addressing the specific needs of this age group and promoting positive change in eating behavior. It is crucial to continue investing in research and development in this field to expand our understanding and optimize intervention strategies, in order to ensure a healthier future for younger generations.

In conclusion, our intervention is situated within a well-established research context that supports the effectiveness of food education and nutritional literacy among adolescents. The addition of digital elements and focus on self-regulation strategies represent significant evolutions in intervention design, offering new directions for future research and educational practice. The convergence between the results of the intervention and those of previous studies strengthens the argument for continuous and innovative investments in food education and health promotion among adolescents.

## Data Availability

The raw data supporting the conclusions of this article will be made available by the authors, without undue reservation.
